# Exogenous Abscisic Acid Can Influence Photosynthetic Processes in Peas through a Decrease in Activity of H^+^-ATP-ase in the Plasma Membrane

**DOI:** 10.3390/biology9100324

**Published:** 2020-10-04

**Authors:** Lyubov Yudina, Ekaterina Sukhova, Oksana Sherstneva, Marina Grinberg, Maria Ladeynova, Vladimir Vodeneev, Vladimir Sukhov

**Affiliations:** Department of Biophysics, N.I. Lobachevsky State University of Nizhny Novgorod, Nizhny Novgorod 603950, Russia; lyubovsurova@mail.ru (L.Y.); n.catherine@inbox.ru (E.S.); sherstneva-oksana@yandex.ru (O.S.); mag1355@yandex.ru (M.G.); ladeynova.m@yandex.ru (M.L.); v.vodeneev@mail.ru (V.V.)

**Keywords:** abscisic acid (ABA), photosynthesis, photosynthetic heat tolerance, electrical signals, variation potential, photosynthetic regulation, CO_2_ assimilation, H^+^-ATP-ase

## Abstract

**Simple Summary:**

Numerous stressors (drought, low and high temperatures, mechanical damages, etc.) act on plants under environmental conditions, suppressing their physiological processes (in particular, photosynthesis). Abscisic acid (ABA) is an important hormone, which participates in increasing plant tolerance to the action of stressors; as a result, treatment by exogenous ABA is a perspective way of regulating the tolerance in agriculture. We investigated the influence of ABA spraying on photosynthetic processes, as well as on their heat tolerance and their regulation by electrical signals propagating after local burning and modifying photosynthesis. It was shown that ABA spraying decreased photosynthetic activity and increased photosynthetic heat tolerance; additionally, the ABA treatment weakened the influence of electrical signals on photosynthesis. We revealed that these responses could be caused by a decrease in activity of H^+^-ATP-ase, which is an important ion transporter in plant cell plasma membrane that supports efflux of H^+^ from cytoplasm. As a whole, our results show the potential influence of the ABA treatment on photosynthetic processes, which is related to a decrease in activity of H^+^-ATP-ase. The result can be potentially useful for development of new methods of management of plant tolerance in agriculture.

**Abstract:**

Abscisic acid (ABA) is an important hormone in plants that participates in their acclimation to the action of stressors. Treatment by exogenous ABA and its synthetic analogs are a potential way of controlling the tolerance of agricultural plants; however, the mechanisms of influence of the ABA treatment on photosynthetic processes require further investigations. The aim of our work was to investigate the participation of inactivation of the plasma membrane H^+^-ATP-ase on the influence of ABA treatment on photosynthetic processes and their regulation by electrical signals in peas. The ABA treatment of seedlings was performed by spraying them with aqueous solutions (10^−5^ M). The combination of a Dual-PAM-100 PAM fluorometer and GFS-3000 infrared gas analyzer was used for photosynthetic measurements; the patch clamp system on the basis of a SliceScope Pro 2000 microscope was used for measurements of electrical activity. It was shown that the ABA treatment stimulated the cyclic electron flow around photosystem I and decreased the photosynthetic CO_2_ assimilation, the amplitude of burning-induced electrical signals (variation potentials), and the magnitude of photosynthetic responses relating to these signals; in contrast, treatment with exogenous ABA increased the heat tolerance of photosynthesis. An investigation of the influence of ABA treatment on the metabolic component of the resting potential showed that this treatment decreased the activity of the H^+^-ATP-ase in the plasma membrane. Inhibitor analysis using sodium orthovanadate demonstrated that this decrease may be a mechanism of the ABA treatment-induced changes in photosynthetic processes, their heat tolerance, and regulation by electrical signals.

## 1. Introduction

The hormonal system plays a key role in the life of higher plants, e.g., phytohormones can regulate their productivity and tolerance to the action of stressors, representing opportunities for using hormonal treatment in agriculture. An important group of hormones is the stress phytohormones, which participate in plant acclimation to the action of adverse factors [1,2], in particular, abscisic acid (ABA). It is known that ABA production can be stimulated by the action of various stressors, including drought [3,4], salinization [4,5], nutrient deficiency [6], different local damages [7,8,9], and others. There are numerous physiological responses, which are caused by changes in the ABA concentration. In particular, ABA activates genes participating in plant acclimation to adverse factors [5], closes stomata and suppresses transpiration [4,10], changes the synthesis of plasma membrane aquaporins [11], influences plant growth [4,6,12,13], activates antioxidant enzymes [14], etc. It is important that participation of ABA in plant adaptation responses to the action of stressors is related to interactions between the ABA signaling pathway and pathways of other stress phytohormones [15,16,17]; i.e., these responses can be achieved through complex regulatory networks involving hormonal crosstalk with ABA. In particular, ABA antagonistically interacts with cytokinins at drought or osmotic stresses participating in plant adaptation [15,16]: ABA represses isopentenyl transferase genes and decreases concentration of cytokinins under drought; in contrast, cytokinins suppress activity of SnRK2 protein kinases, which are key enzymes in induction of ABA-induced responses, under favorable conditions. Brassinosteroids are another group of the stress phytohormones interacting with the ABA signaling because they regulate the plant drought adaptation through signaling components linked to the ABA signaling pathway [17].

Photosynthesis is the basis of plant productivity and participates in tolerance to stressors. Its activity has both positive and negative controls through regulation of CO_2_ entry by stomata [18]; e.g., stomata can be opened by decreased concentration of CO2 [18,19,20,21] or high intensity light [21] and closed by increased concentration of CO_2_ [18,22,23] or development of vapor pressure deficit [21,23,24]. Some phytohormones, namely indole-3-acetic acid [18] and cytokinins [18,25,26,27], participate in the stomata opening; in particular, cytokinins eliminate the stomata closing induced by increased CO_2_ concentration [18]. In contrast, ABA is known to induce the stomata closing [18,25]; i.e., it can be expected that changes in ABA concentration are a mechanism of the negative control of photosynthesis.

There are a number of works which have investigated the influence of exogenous ABA treatment on photosynthetic processes [28,29,30,31,32,33]. It was shown that the treatment rapidly decreases photosynthetic CO_2_ assimilation (A_CO2_) under light conditions and can increase the cyclic electron flow around photosystem I [29,30,31,32,33]. The changes are considered to be caused by a decrease in CO_2_ flux into the stroma of chloroplasts because the maximal rate of CO_2_ fixation [32,34] and the compensation value of the intercellular CO_2_ concentration [31,32,33] do not seem to change. The CO_2_ flux decrease can be caused by stomata closing [32,34]; the ABA treatment-induced decrease in the intercellular CO_2_ concentration [29] and the weak ABA influence on the CO_2_ assimilation in mutants, which has been shown to impair regulation of the stomatal closure by ABA [30], support this mechanism. However, an alternative mechanism of the ABA treatment-induced decrease in photosynthetic activity may be related to a decrease in the plasma membrane conductance for CO_2_ [33]; the decrease influences the CO_2_ leaf mesophyll conductance as a whole. It is important that the latter mechanism possibly plays a key role after plant spraying with moderate concentrations of exogenous ABA (e.g., 10^−6^–10^−5^ M) [33], because changes in the stomata conductance are weak at these concentrations.

Previously, we hypothesized that an ABA treatment-induced decrease in activity of H^+^-ATP-ase in the plasma membrane is an important mechanism of photosynthetic regulation [33]. There are some arguments supporting this hypothesis. First, ABA decreases the activity of this transporter [12,33,35], which may be related to Ca^2+^ flux into the cytoplasm [3,36], suppressing the activity of the H^+^-ATP-ase [37,38,39,40]. Secondly, the activity of the H^+^-ATP-ase and values of extra- and intracellular pH, which are dependent on this activity, can strongly influence photosynthetic processes, including A_CO2_ [41,42,43,44]; in particular, this effect can be caused by pH-dependent changes in the CO_2_ to HCO_3_^–^ ratio in apoplasts [42,44,45] because the uncharged form can pass through the lipid membranes much more easily than the charged one [45,46]. Alternatively, a decrease in the intracellular pH, which is related to H^+^-ATP-ase inactivation [41,42,47], can also influence photosynthetic light reactions, including changes in the localization of ferredoxin-NADP(H) oxidoreductase [48,49]; stimulate the cyclic electron flow around photosystem I [50]; increase non-photochemical quenching of the chlorophyll fluorescence (NPQ) [51,52]; probably modify the distribution of light energy between photosystem I and II [53], etc. Some of these effects can be observed after ABA treatment (e.g., activation of the cyclic electron flow [33]).

However, there are some questions, which are related to the hypothesis about the participation of H^+^-ATP-ase inactivation in the response induced by exogenous ABA treatment. First, can a decrease in the H^+^-ATP-ase activity, which is induced by other factors, influence photosynthetic processes in a similar manner to the ABA effect? Can this decrease modify the ABA treatment-induced photosynthetic changes?

Second, ABA is known to modify the plant tolerance to the action of stressors [1,2], i.e., the problem of the participation of the decrease in H^+^-ATP-ase activity in the ABA-induced changes in plant tolerance to stressors also requires experimental analysis. In particular, the decrease in A_CO2_, activation of the cyclic electron flow around photosystem I (CEF), and increase in the ATP content in plants (i.e., changes which can be caused by a decrease in the H^+^-ATP-ase activity and shifts in intra- and extracellular pH [38,39,40]) participate in the increase in the photosynthetic tolerance to moderate heating [37,40,54,55], which is an important environmental stressor. It is known [56,57,58] that ABA treatment can increase a plant’s thermal tolerance, including its photosynthetic tolerance. Therefore, it can be potentially expected that the ABA treatment-induced decrease in the H^+^-ATP-ase activity can participate in the increase in the photosynthetic tolerance to moderate heating.

Third, inactivation of the H^+^-ATP-ase participates in the generation of electrical signals (especially variation potentials, VPs) [37,39,59,60] and, probably, in the formation of various fast physiological responses, induced by these signals [38,40]. In particular, it is known that electrical signals interact with hormonal signaling [61,62] (including the ABA signal [8,9,63]), strongly influence photosynthetic processes [42,50,53,59,60,64,65,66,67], change transpiration [41,64], increase the plant tolerance to stressors [55,68,69,70,71,72], and induce many other responses (e.g., stimulation of the expression of defense genes [66,73], activation of respiration [74], and suppression of phloem mass flow [75,76]). Many of these responses are similar to responses induced by ABA treatment; as a result, it can be expected that the exogenous ABA can modify electrical signals and physiological responses caused by these signals. However, this supposition requires experimental investigations.

Therefore, the aim of our work was to experimentally investigate the influence of exogenous ABA treatment on photosynthesis, photosynthetic heat tolerance, and regulation by electrical signals in peas, including an analysis of the participation of the decrease in the H^+^-ATP-ase activity in these changes.

## 2. Materials and Methods

### 2.1. Plant Materials and Treatments

Two to three-week-old pea seedlings (*Pisum sativum* L.) were used in this investigation. Plants were cultivated hydroponically (a half-strength Hoagland–Arnon medium) in a Binder KBW 240 climatic chamber (Binder GmbH, Tuttlingen, Germany) at 23 °C under a 16/8 light/dark photoperiod. Small vessels (for 10 seedlings) were used for hydroponic growth. Photosynthetic and electrical parameters were investigated in the second mature leaves.

**Figure 1:**
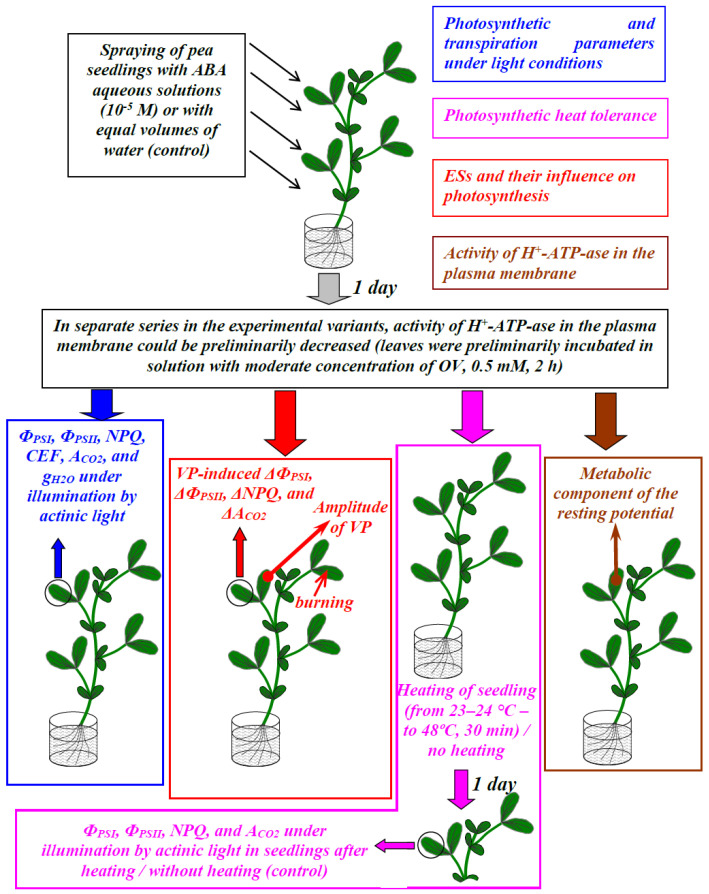
Schemes of different variants of experiments which were used in the current work. Fourteen to 21-day-old pea seedlings were investigated. There were four experimental variants, including an analysis of the exogenous abscisic acid (ABA) influence on (i) photosynthetic parameters under illumination by the blue actinic light (460 nm, 239 µmol m^−2^s^−1^); (ii) photosynthetic heat tolerance; (iii) changes in photosynthetic parameters and electrical activity, induced by local burning; (iv) the metabolic component of the resting potential, which was related to the activity of H^+^-ATP-ase in the plasma membrane. In separate series of the experimental variants, the activity of H^+^-ATP-ase in the plasma membrane could be preliminarily decreased (leaves were preliminarily incubated in water solution with a moderate concentration of sodium orthovanadate ((OV), 0.5 mM, 2 h); this treatment was used for imitating the influence of exogenous ABA and for the modification of ABA-induced photosynthetic changes. Measurement of the metabolic component of the resting potential was based on fast inactivation of the H^+^-ATP-ase (minutes) under the action of the high concentration of OV (5 mM); OV was added into solution, which was placed in contact with investigated plant cells, and changes in electrical potential were measured. Local burning of the first mature leaf was induced by a flame (3–4 s, about 1 cm^2^) [42,43,47,50,53,55]; we did not analyze parameters of propagation of burning-induced electrical signals (variation potential, VP) in detail. Photosynthetic A_CO2_ was calculated as the difference between the CO_2_ assimilation rate before the termination of illumination by the actinic light (after 10 min of illumination) and this rate 5 min after the termination (after 5 min of dark conditions).

The exogenous ABA treatment (Sigma-Aldrich, St. Louis, MO, USA) of seedlings was performed by spraying them with ABA aqueous solutions (Figure 1), in accordance with the widely used method of ABA treatment [14,20]. The spraying was continued up to full wetting of the leaves (about 20 mL per vessel). The ABA concentration in the solution was 10^–5^ M, in accordance with our previous work [33]. Control plants were treated with similar volumes of distilled water. The ABA treatment was performed 1 day before the initiation of other experimental procedures because we previously showed that a decrease in photosynthesis in pea seedlings was observed 1 day after the ABA treatment and was absent 2 or 3 days after that [33].

In the experimental series, we preliminarily treated the second mature leaves with a moderate concentration of sodium orthovanadate (OV, Sigma-Aldrich, St. Louis, MO, USA). OV suppresses the activity of P-type transport ATP-ases; in particular, it decreases the activity of H^+^-ATP-ase [68,77], which is the main active electrogenic transporter in the plasma membrane of cells of higher plants. In accordance with our previous work [68], the preliminary treatment was performed by incubation of the second leaves in a water solution of OV (0.5 mM OV + standard solution, including 1 mM KCl, 0.1 mM NaCl and 0.5 mM CaCl_2_) for 2 h; after that, the solution was eliminated from the leaf surface (leaves were dried by filter paper). Leaves of control plants were incubated in the standard solution. Experimental procedures started 10 min after termination of the incubation.

### 2.2. Measurements of Photosynthesis under Light Conditions

A standard system (Heinz Walz GmbH, Effeltrich, Germany), which included a GFS-3000 gas analyzer, Dual-PAM-100 PAM-fluorometer, and Dual-PAM gas-exchange Cuvette 3010-Dual common measuring head, was used for photosynthetic investigations. The concentration of CO_2_ and the relative humidity in the measuring cuvette were 360 ppm and about 70%, respectively; the temperature of the investigated leaf was constant (23 °C). Dual-PAM-100 was used for the illumination of leaves by different types of light. The intensity of the measuring blue light (460 nm) was 24 µmol m^−2^s^−1^. The intensity of red saturation pulses (630 nm, 300 ms) was 10,000 µmol m^−2^s^−1^. The intensity of the blue actinic light (460 nm) was 240 µmol m^−2^s^−1^.

Measurements of parameters of photosynthetic light reactions started after a 10 min dark interval [55,68]. Parameters of light photosynthetic reactions were measured using Dual-PAM-100. First, the initial and maximal levels of photosystem II fluorescence (*F_0_* and *F_m_*, respectively) and maximal light adsorption by photosystem I (*P_m_*) were measured. After that, leaves were illuminated by the actinic light for 10 min. Saturation pulses were generated every 10 s and the current levels of fluorescence (*F*), maximal fluorescence level after the preliminary illumination (*F_m_*'), current light adsorption by photosystem I (*P*), and maximal light adsorption by photosystem I after the preliminary illumination (*P_m_'*) were measured. Parameters of photosynthetic light reactions, including the effective quantum yields of photosystem I (Φ_PSI_) and II (Φ_PSII_) and NPQ, were calculated for every saturation pulse on the basis of the measured parameters, in accordance with standard equations [78,79]. We investigated the last Φ_PSI_, Φ_PSII_, and NPQ, which were measured before the termination of illumination (after 10 min of illumination), for an analysis of the influence of exogenous ABA treatment on photosynthetic parameters under light conditions.

We used a previously described method [33,50] for the calculation of the cyclic electron flow around photosystem I (CEF). In accordance with Equation (1), CEF was calculated as

(1) $$\mathrm{CEF}=p\times \mathrm{PAR}\times \left(dII\times \Phi_{PSII}-\left(1-\mathrm{dII}\right)\times \Phi_{PSI}\right)$$

where p is a part of the total flux of the photosynthetically active radiation (PAR), which was absorbed by the leaf; p was 0.88 for peas, in accordance with our earlier work [33]. dII is the part of the absorbed radiation that was distributed to photosystem II. We calculated dII in accordance with Equation (2) [50,53] for each CEF calculation of individual seedlings:

(2) $$dII=\frac{\Phi_{PSI}}{\Phi_{PSI}+\Phi_{PSII}}$$

where Φ_PSI_ and Φ_PSII_ were measured under a low intensity of PAR (about 24 µmol m^−2^s^−1^).

GFS-3000 was used for measurements of A_CO2_ and the leaf water conductance (g_H2O_), which were automatically calculated by the GFS-3000 software.

### 2.3. Estimation of the Photosynthetic Heat Tolerance

We used the transient heating of whole pea seedlings for an investigation of their photosynthetic heat tolerance, in accordance with our preliminarily works [68,80]. In the current experiments, pea seedlings were heated in a TV-20-PZ-“K” thermostat (Kasimov Instrument Plant, Kasimov, Russia) from between 23 and 24 °C to 48 °C for 30 min; in control variants, heating was absent. Heating was performed 1 day after the exogenous ABA treatment. A_CO2_ and the parameters of photosynthetic light reactions in leaves were measured 1 day after heating; the experimental procedure was similar to the procedure described in Section 2.2.

### 2.4. Investigation of Electrical Signals and Photosynthetic Responses Induced by These Signals

The patch clamp system, on the basis of a SliceScope Pro 2000 microscope (Scientifica, Uckfield, UK), was used for intracellular measurements of the electrical activity of pea seedlings. The system included a motorized microscope and micromanipulators mounted on a vibration-proof table protected by a Faraday shield, MultiClamp 700B amplifier (Molecular Devices, San Jose, CA, USA), DIGIDATA 1550 data acquisition system (Molecular Devices), and personal computer (PC). Micropipettes (tip diameter was below 1 µm, resistance was about 40 MOhm) were fabricated on a P-97 Sutter Micropipette Puller (Sutter Instrument, Novato, CA, USA). Measuring microelectrodes were filled with 0.1 M KCl and were inserted into cells of mesophyll parenchyma of the second mature pea leaves. Chlorinated silver wire was used as the reference electrode and was immersed into the solution in the experimental chamber.

In a separate series of experiments (investigation of the ABA treatment’s influence on electrical signal-induced photosynthetic responses), we also measured electrical signals on the basis of changes in the surface electrical potential by using extracellular measurements. The method was simpler than intracellular measurements of electrical activity; extracellular measurements and photosynthetic measurements could be simultaneously performed in the same leaf (in different leaflets, e.g., see [50]). The surface electrical potential was measured using Ag^+^/AgCl electrodes (Gomel Measuring Equipment Plant, Gomel, Belarus), a high-impedance IPL-113 amplifier (Semico, Novosibirsk, Russia), and a PC. The electrode was connected to the center of the leaflet of the investigated leaf, and the reference electrode was placed in solution surrounding the root.

Local burning of the upper part of the first mature leaf was induced by an open flame; the duration of burning was 3–4 s and the size of the damaged zone was about 1 cm^2^. The local burning was used for the induction of electrical signals, in accordance with our previous work with pea seedlings [42,43,47,50,53,55]. It should be noted that local burning is a widely used stimulation for the induction of electrical signals; mainly, variation potential (VP) [37], which is a damage-induced depolarization signal in higher plants with a long duration and variable shape. We did not investigate the parameters of propagation of VP in the current work; only microelectrode and extracellular measurements of variation potentials in the second leaves were performed. The induction of VP occurred 1.5 h after the fixation of seedlings in the measuring system.

Measurements of local burning-induced photosynthetic responses, which were related to the generation and propagation of VP, were described in detail in our previous works [42,43,47,50,53,55]. As a whole, the photosynthetic measurements were similar to measurements described in Section 2.2; however, the duration of illumination by actinic light and measurements of A_CO2_ and parameters of photosynthetic light reactions were about 2 h (1.5 h after plant fixation and about 20–30 min of measurement of fast photosynthetic changes after VP induction by burning).

### 2.5. Estimation of the Metabolic Component of the Resting Potential

The metabolic component of the resting potential can be used for an estimation of H^+^-ATP-ase activity in the plasma membrane [33] because this transporter is the main mechanism of the active transport of ions in higher plants [81,82]. We measured the metabolic component on the basis of the amplitude of the fast depolarization of the membrane potential (several minutes) after the injection of OV into solution in the experimental chamber, in accordance with our previous work [33]. The high concentration of OV (final concentration of OV was 5 mM) suppressed H^+^-ATP-ase activity, i.e., the difference between the initial value of the membrane potential and its maximal value after OV injection showed the magnitude of the metabolic component of the resting potential.

### 2.6. Statistics

Different seedlings of peas were used for each experiment. Quantities of repetitions were varied in different experimental variants and are shown in the figures. Mean values, standard errors, representative records, scatter plots, and correlation coefficients are shown in the figures. The significance of differences was estimated using the Student’s t-test. The standard functions of Microsoft Excel were used for statistical analysis.

## 3. Results

### 3.1. Investigation of Changes in Photosynthesis, Photosynthetic Heat Tolerance, and Regulation by Electrical Signals after Treatment with Exogenous ABA

**Figure 2:**
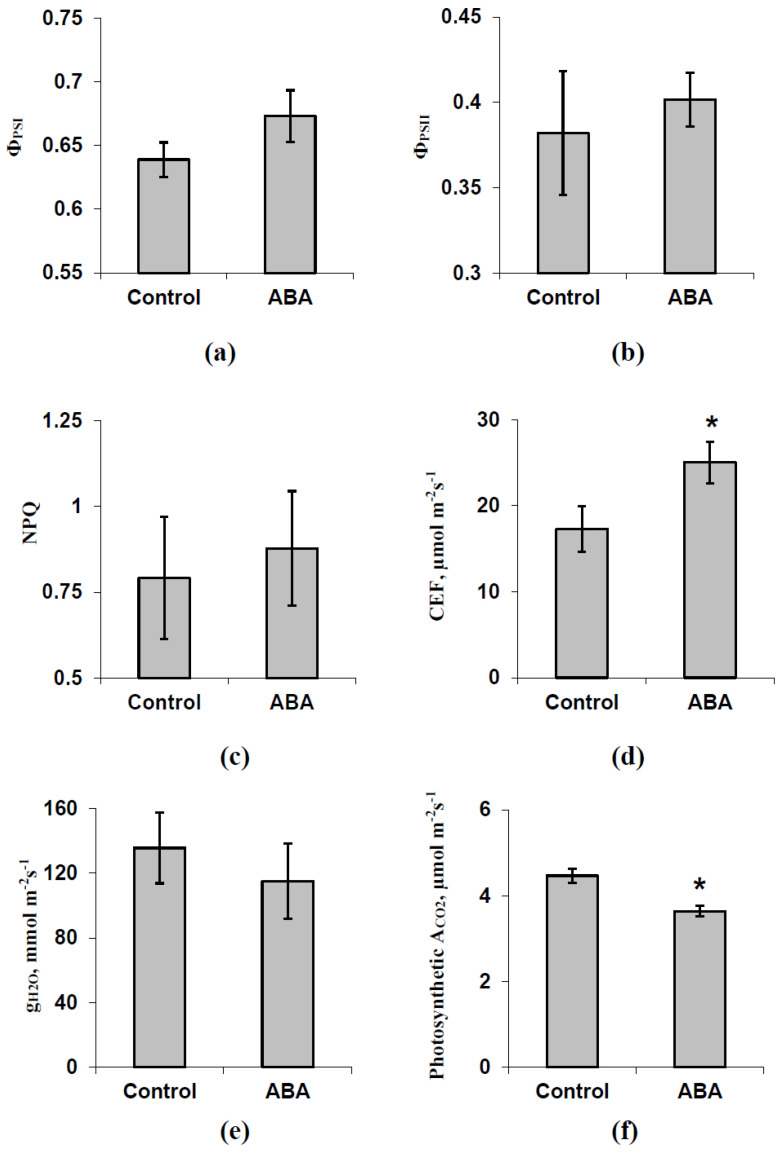
Quantum yields of photosystems I (Φ_PSI_) (**a**) and II (Φ_PSII_) (**b**), the non-photochemical quenching of chlorophyll florescence (NPQ) (**c**), the cyclic electron flow around photosystem I (CEF) (**d**), the leaf water conductance (g_H2O_) (**e**), and the photosynthetic assimilation of CO_2_ (A_CO2_) (**f**) after the ABA treatment in pea seedlings (*n* = 5-15). The ABA treatment of seedlings was performed by spraying them with aqueous solutions (10^−5^ M) 1 day before photosynthetic measurements; control plants were treated with equal volumes of water. Photosynthetic parameters and leaf water conductance were measured after 10 min of illumination by blue actinic light (239 µmol m^−2^s^−1^) in the second mature leaf. *, difference between experiment and control plants is significant (*p* < 0.05).

The analysis of the influence of the exogenous ABA treatment (spraying) on photosynthetic processes under light conditions showed that the investigated parameters of the light reactions (Φ_PSI_, Φ_PSII_, and NPQ) in pea leaves were not affected by this treatment (Figure 2). Only a weak and insignificant increase in Φ_PSI_ was observed in treated pea seedlings. In contrast, CEF was significantly increased after the ABA treatment; the relative magnitude of the increase was about 47%. Significant changes in g_H2O_ were absent. Photosynthetic A_CO2_ was significantly decreased after the ABA treatment; the relative magnitude of this decrease was about 20%. It was interesting that the magnitudes of changes in CEF and A_CO2_ were negatively related (R = −0.70, *p* < 0.05). The results were in good accordance with our previous data [33], which showed an ABA-induced decrease in CO_2_ assimilation and increase in CEF; however, changes in Φ_PSI_, Φ_PSII_, and g_H2O_ were absent.

**Figure 3:**
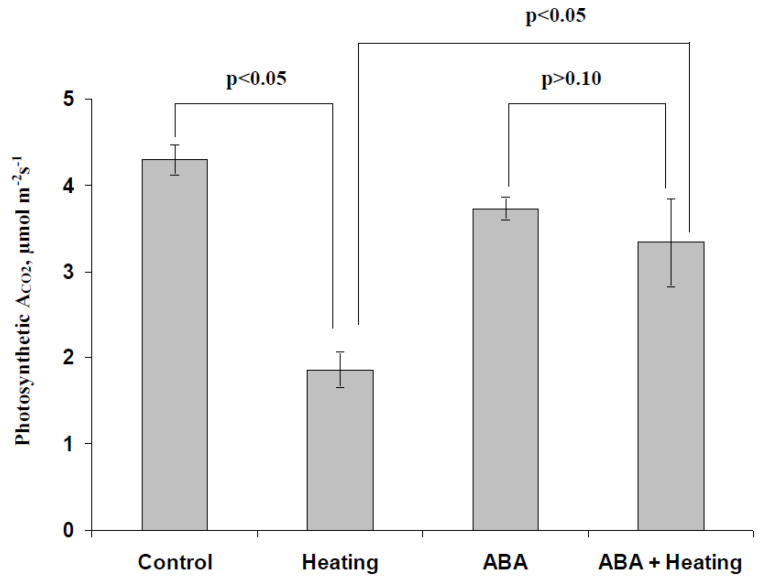
Influence of the ABA treatment on the photosynthetic assimilation of CO_2_ (A_CO2_) after heating in pea seedlings (*n* = 5–15). The ABA treatment of seedlings was performed by spraying them with aqueous solutions (10^−5^ M) 1 day before heating; plants without the ABA treatment were treated with equal volumes of water. Seedlings were heated from between 23 and 24 °C to 48 °C for 30 min using a thermostat. The photosynthetic CO_2_ assimilation was measured after 10 min of illumination by blue actinic light (239 µmol m^−2^s^−1^); photosynthetic measurements were performed 1 day after heating in the second mature leaf.

Figure 3 shows that the pea seedling heating induced a significant decrease in photosynthetic A_CO2_ in leaves; the relative magnitude of this decrease 1 day after heating was about 57%. The exogenous ABA treatment, which was performed 1 day before the heating of plants, increased the CO_2_ assimilation, which was significantly higher than this assimilation after heating in seedlings without spraying by ABA. Moreover, a significant difference between ABA-treated seedlings under control conditions and ones after heating was absent, i.e., heating did not induce a strong suppression of photosynthetic A_CO2_ in peas after treatment with the exogenous ABA. It should be noted that heating also induced a significant decrease in Φ_PSII_ in pea leaves (significant changes in Φ_PSI_ and NPQ were absent); however, the difference between photosystem II quantum yields in the heated plants with the ABA treatment and ones without this treatment was weak and insignificant (data not shown). These results showed that treatment with the exogenous ABA can increase the heat tolerance of photosynthetic CO_2_ assimilation in pea seedlings; this effect is probably relatively late onset because the tolerance increase was observed 1 day after the ABA treatment.

**Figure 4:**
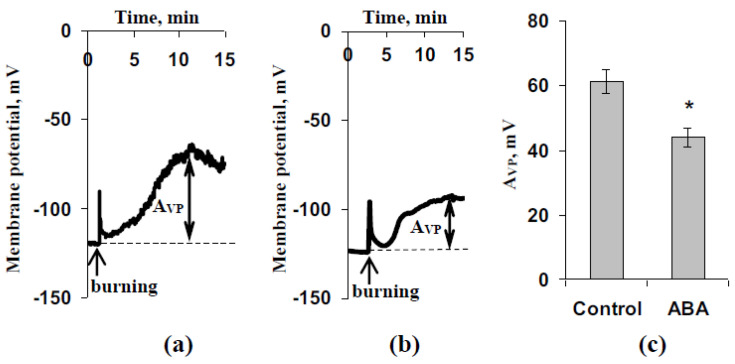
(**a**) Microelectrode record of burning-induced variation potential (VP) in the leaf of a control pea seedling. (**b**) Microelectrode record of burning-induced variation potential (VP) in the leaf of a seedling 1 day after the ABA treatment. **(c)** Average amplitudes of VP in control seedlings and seedlings after the ABA treatment (*n* = 5). The ABA treatment of seedlings was performed by spraying them with aqueous solutions (10^−5^ M) 1 day before electrical measurements; control plants were treated with equal volumes of water. Electrical measurements were performed in the second mature leaf. Variation potentials were induced (burning of the first mature leaf by a flame, 3-4 s, about 1 cm^2^) 1.5 h after plant fixation for measurement. A_VP_ was calculated as the difference between maximal and initial values of the membrane potential. *, difference between experiment and control plants is significant (*p* < 0.05).

Figure 4 shows typical electrical signals, which were observed in plants after the ABA treatment and without this treatment. The signal was measured in the mesophyll cell in the second mature leaf of pea seedlings after local burning of the first leaf. The electrical signal had a long duration (more than 10 min) and complex dynamics of changes in the membrane potential (including a fast depolarization spike and slow, long-lasting depolarization after that), i.e., it can be classified as VP [37,83,84]. The average amplitude of VP without the exogenous ABA treatment was about 60 mV (Figure 4c). The ABA treatment 1 day before the measurements of electrical activity significantly decreased the amplitude of VP (from 60 mV to about 44 mV). Therefore, the relative decrease in amplitude VP in the second pea leaf after the ABA treatment was about 28%. VPs were also measured by using extracellular electrical measurements (Supplementary Materials, Figure S1); these measurements supported the significant decrease in the VP amplitude after the ABA treatment.

**Figure 5:**
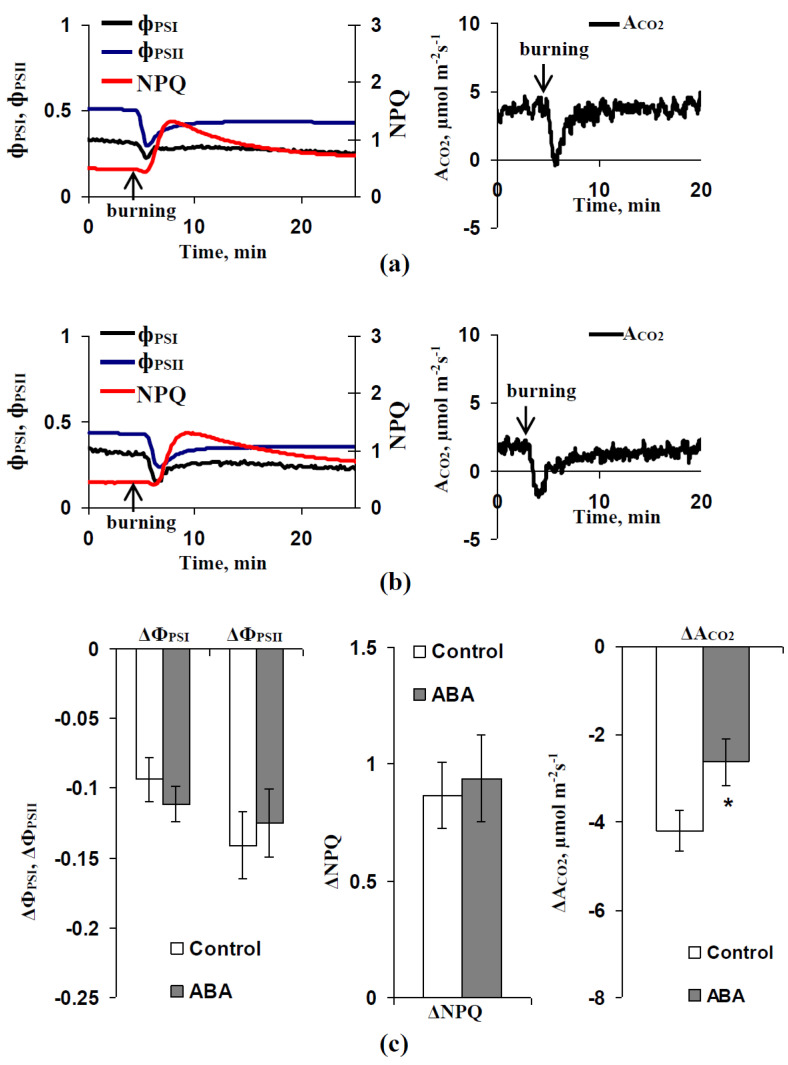
(**a**) Changes in quantum yields of photosystems I (Φ_PSI_) and II (Φ_PSII_), the non-photochemical quenching of chlorophyll florescence (NPQ), and the photosynthetic assimilation of CO_2_ (A_CO2_) in the leaf of a control pea seedling after local burning. (**b**) Changes in quantum yields of photosystems I (Φ_PSI_) and II (Φ_PSII_), the non-photochemical quenching of chlorophyll florescence (NPQ), and the photosynthetic assimilation of CO_2_ (A_CO2_) in the leaf of an ABA-treated pea seedling after local burning. **(c)** Average magnitudes of these changes in photosynthetic parameters in control seedlings and seedlings after the ABA treatment (*n* = 5-7). The ABA treatment of seedlings was performed by spraying them with aqueous solutions (10^−5^ M) 1 day before photosynthetic measurements; control plants were treated with equal volumes of water. Photosynthetic measurements were performed in the second mature leaf; illumination by blue actinic light (239 µmol m^−2^s^−1^) was used. Local burning of the first mature leaf by a flame (3-4 s, about 1 cm^2^) was performed 1.5 h after plant fixation for measurement. *, difference between experiment and control plants is significant (*p* < 0.05).

Figure 5 shows the typical records of the changes in the CO_2_ assimilation and parameters of photosynthetic light reactions, which were induced by local burning of the first leaf, in the second leaf of the pea seedlings after exogenous ABA treatment and without this treatment. The local burning caused transient decreases in A_CO2_, Φ_PSI_, and Φ_PSII_; in contrast, NPQ was increased after that. It should be noted that this response included fast changes in photosynthetic parameters, which had extremes of about 2–5 min after the local burning. The maximal magnitudes of these fast changes were further investigated.

Figure 5c shows the average magnitudes of these changes in pea leaves in seedlings, which were treated by the exogenous ABA and were not treated. It was shown that the ABA treatment weakly influenced the magnitudes of the local burning-induced decrease in Φ_PSI_ and Φ_PSII_ and increase in NPQ; all differences between changes in these parameters of photosynthetic light reactions in treated and untreated plants were insignificant. In contrast, the magnitude of the local burning-induced suppression of A_CO2_ was significantly decreased after the ABA treatment (by approximately 37%).

It was important that these photosynthetic responses were typical responses observed in higher plants [38] (including peas [42,47,50,55,69]) after local damage and caused by VP propagation. Figure S1d (Supplementary Materials) supports the participation of VP in the induction of these photosynthetic changes, because their individual magnitudes were linearly correlated with VP amplitudes; the correlation was significant.

The decrease in magnitude of the local burning-induced response of CO_2_ assimilation after the ABA treatment could be related to the decrease in the amplitude of the variation potential, which was induced by local burning, because these values could be linearly correlated (current results and [53,85]). However, this decrease may also have been caused by the decrease in the rate of photosynthetic CO_2_ assimilation before the induction of VP in pea seedlings, which were preliminarily treated with the exogenous ABA. If the last hypothesis is correct, it can be expected that the initial rate of A_CO2_ (before the induction of VP) is strongly related to the magnitudes of local burning-induced decreases in CO_2_ assimilation.

**Figure 6:**
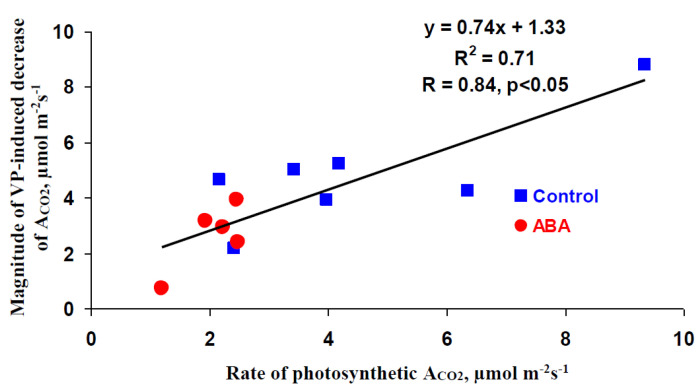
A scatter plot showing the rates of photosynthetic CO_2_ assimilation (A_CO2_) and magnitudes of local burning-induced decreases in A_CO2_ in pea seedlings (*n* = 12). R^2^ and R are determination and correlation coefficients, respectively.

Figure 6 shows a scatter plot between initial rates of the photosynthetic CO_2_ assimilation and magnitudes of local burning-induced assimilation responses. It was shown that these values were strongly linearly related. The value of the determination coefficient was 0.71; the correlation coefficient was equal to 0.84 and was significant. Therefore, the decrease in the rate of photosynthetic CO_2_ assimilation under light conditions, which was observed 1 day after the exogenous ABA treatment, could also participate in the decrease in the magnitude of the local burning-induced photosynthetic response in the ABA-treated pea seedlings. It is interesting that a significant difference between relative magnitudes of local burning-induced changes in A_CO2_ (ratio of the magnitude of the local burning-induced A_CO2_ decrease to the rate of A_CO2_ before local burning) in seedlings with the ABA treatment and ones without this treatment was absent (data not shown).

### 3.2. Influence of the ABA and Sodium Orthovanadate Treatment on the Metabolic Component of the Resting Potential

Figure S2 shows that the injection of OV into solution surrounding investigated cells (the final OV concentration was high and equal to 5 mM) induced fast depolarization of the plasma membrane potential, which was caused by the inhibition of H^+^-ATP-ase. It was interesting that the dynamics of the membrane potential change were similar to the dynamics of membrane potential change during the generation of VP (see Figure 4 or examples in [24]); this similarity was possibly based on the participation of strong inactivation of the plasma membrane H^+^-ATP-ase in both processes. The magnitude of the fast OV-induced depolarization (in the range of minutes) was used for an estimation of the metabolic component of the resting potential, i.e., for an estimation of the H^+^-ATP-ase activity under control conditions or after treatments by the exogenous ABA and a moderate concentration of OV.

**Figure 7:**
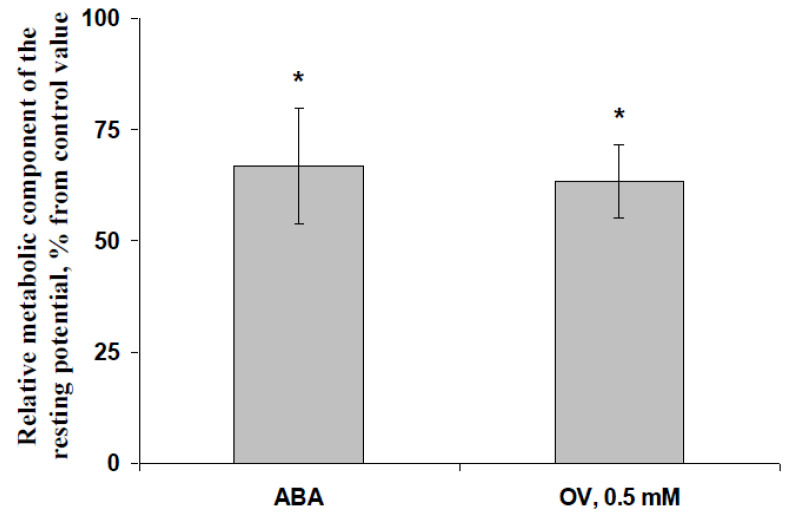
Relative values of the metabolic component of the resting potential in pea seedlings after treatment by ABA and a moderate concentration of sodium orthovanadate (OV) (*n* = 5–7). The ABA treatment of seedlings was performed by spraying them with aqueous solutions (10^−5^ M) 1 day before electrical measurements; control plants were treated with equal volumes of water. The preliminary treatment with the moderate OV concentration in the second mature leaf in seedlings was performed by incubation of the leaf (2 h) in a solution of OV (0.5 mM); after that, this leaf was dried by filter paper and was used for the measurement of electrical activity. Similar treatment by water was used in the control. Measurements of the metabolic component of the resting electrical potential across the plasma membrane were performed with the addition of a high concentration of OV (5 mM) during the electrical record; only short-term changes in the membrane potential were analyzed (see Figure S2 for details). The metabolic component was formed by the active transport of H^+^ across the plasma membrane, i.e., it was strongly related to H^+^-ATP-ase activity in the plasma membrane. Relative values of the metabolic components were calculated as the ratio of experimental values to control ones. *, difference between experiment and control plants is significant (*p* < 0.05).

Figure 7 shows that the exogenous ABA treatment significantly decreased the metabolic component of the resting potential which was measured 1 day after the treatment; the magnitude of this decrease was about 33%. The result was in good accordance with our previous results [33], which showed that the ABA treatment decreased the metabolic component of the resting potential by 20–40%. It was very interesting that the preliminary treatment of the second pea leaves by a moderate concentration of OV (0.5 mM and 2 h of incubation, in accordance with our earlier work [68]) induced a similar decrease in the metabolic component; the magnitude of this decrease was about 37%. Considering the key role of the plasma membrane H^+^-ATP-ase in the formation of the metabolic component [81,82], the ABA treatment-induced decrease in the metabolic component showed a decrease in activity of this H^+^-ATP-ase.

The last result was important for further analysis because it was shown that the preliminary treatment of leaves by a moderate concentration of OV could induce changes in the metabolic potential, which were similar to the ABA treatment-induced changes, i.e., this variant of OV treatment could be used for imitating ABA’s influence on H^+^-ATP-ase in the plasma membrane.

### 3.3. Analysis of the Participation of the Decrease in the H^+^-ATP-ase Activity in the Influence of the ABA Treatment on Photosynthetic Processes and Their Regulation by Electrical Signals

The next stage of the investigation was devoted to an analysis of the participation of the decrease in the H^+^-ATP-ase activity (using preliminary treatment of pea leaves with a moderate concentration of OV [33,68]) in the influence of the exogenous ABA treatment on the photosynthesis under light conditions, photosynthetic heat tolerance, and regulation by electrical signals. Considering the absence of a significant influence of the ABA treatment on the investigated parameters of photosynthetic light reactions in leaves (Φ_PSI_, Φ_PSII_, and NPQ) and significant relation between changes in CEF and A_CO2_, we only analyzed the CO_2_ assimilation in this part of the work.

**Figure 8:**
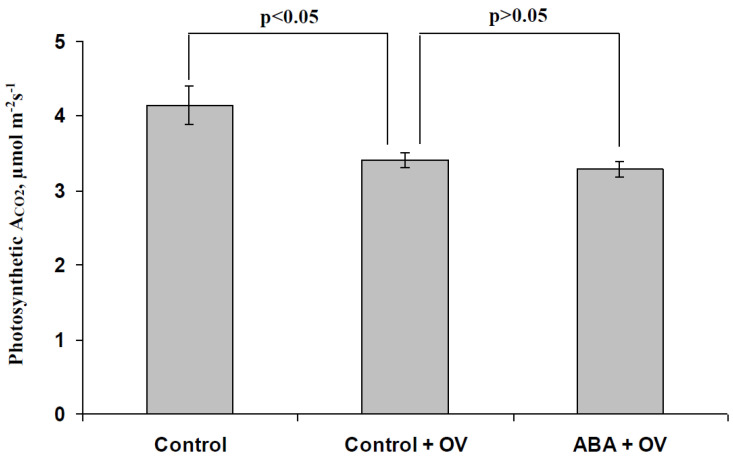
Influence of the modification of the H^+^-ATP-ase activity by sodium orthovanadate (OV) on photosynthetic CO_2_ assimilation after the ABA treatment (*n* = 5–10). The ABA treatment of seedlings was performed by spraying them with aqueous solutions (10^−5^ M) 1 day before photosynthetic measurements; control plants were treated with equal volumes of water. The preliminary OV treatment of the second mature leaf in seedlings was performed by incubation of the leaf (2 h) in a solution of OV with a moderate concentration (0.5 mM); after that, this leaf was dried by filter paper and used for photosynthetic measurements. Similar treatment by water was used in the control. Photosynthetic parameters were measured after 10 min of illumination by blue actinic light (239 µmol m^−2^s^−1^) in the second mature leaf.

Figure 8 shows that a decrease in H^+^-ATP-ase activity decreased A_CO2_ under light conditions; the relative magnitude of this decrease was about 18%. In contrast, significant differences between the rates of photosynthetic CO_2_ assimilation in pea seedlings, which were treated with the moderate OV concentration, and seedlings which were treated with both the moderate OV concentration and exogenous ABA, were absent. These results showed that the decrease in the activity of the H^+^-ATP-ase in the plasma membrane could participate in the influence of the ABA treatment on photosynthesis under light conditions.

**Figure 9:**
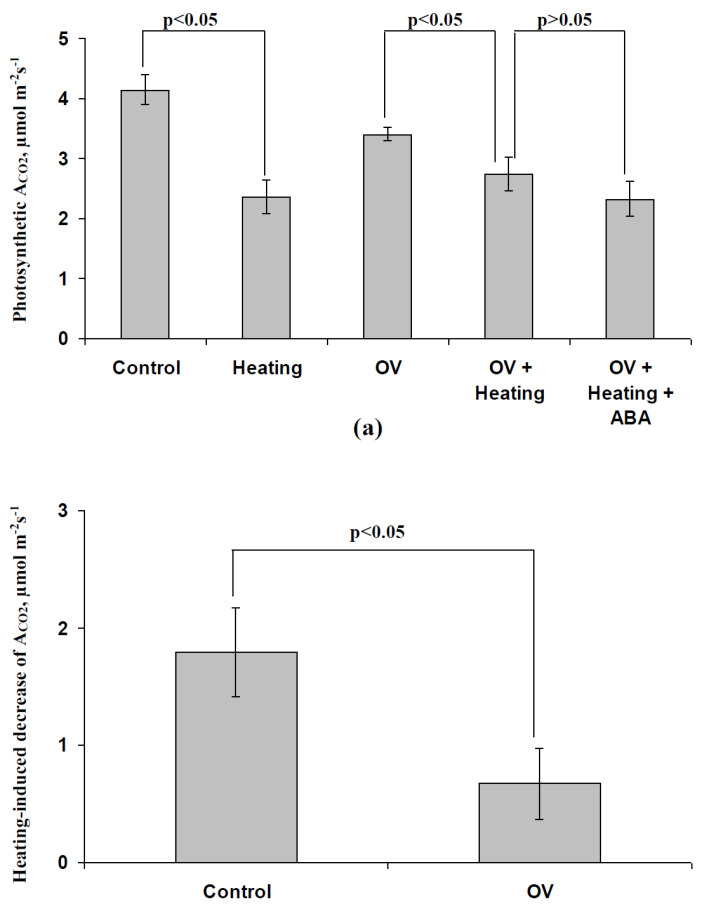
Influence of the modification of H^+^-ATP-ase activity by sodium orthovanadate (OV) on the photosynthetic heating tolerance of pea seedlings in the control group and after the ABA treatment (*n* = 5-14). **(a)** Rates of the photosynthetic CO_2_ assimilation (A_CO2_) in different variants of the experiment. **(b)** Heating-induced decreases in A_CO2_ in control and OV treatment groups. The ABA treatment of seedlings was performed by spraying them with aqueous solutions (10^−5^ M) 1 day before heating; control plants were treated with equal volumes of water. The preliminary OV treatment of the leaves of seedlings was performed by incubation of the leaf (2 h) in a solution of OV with a moderate concentration (0.5 mM); after that, this leaf was dried by filter paper and seedlings were heated. Similar treatment by water was used in the control. Seedlings were heated from 23–24 °C to 48 °C for 30 min using a thermostat. The photosynthetic CO_2_ assimilation was measured after 10 min of illumination by blue actinic light (239 µmol m^−2^s^−1^); photosynthetic measurements were performed 1 day after heating in the second mature leaf.

Figure 9 shows the participation of the decrease in the H^+^-ATP-ase activity in the ABA-induced increase in the photosynthetic heat tolerance. In particular, it was shown that the preliminary treatment with the moderate OV concentration significantly influenced the magnitude of the heating-induced decrease in photosynthetic CO_2_ assimilation (Figure 9b); the relative magnitude of this decrease after the OV treatment was only 37% of that without the OV treatment. Moreover, significant differences between the photosynthetic CO_2_ assimilation after heating in seedlings that were treated with the moderate OV concentration and seedlings that were treated with both the moderate OV concentration and exogenous ABA, were absent.

**Figure 10:**
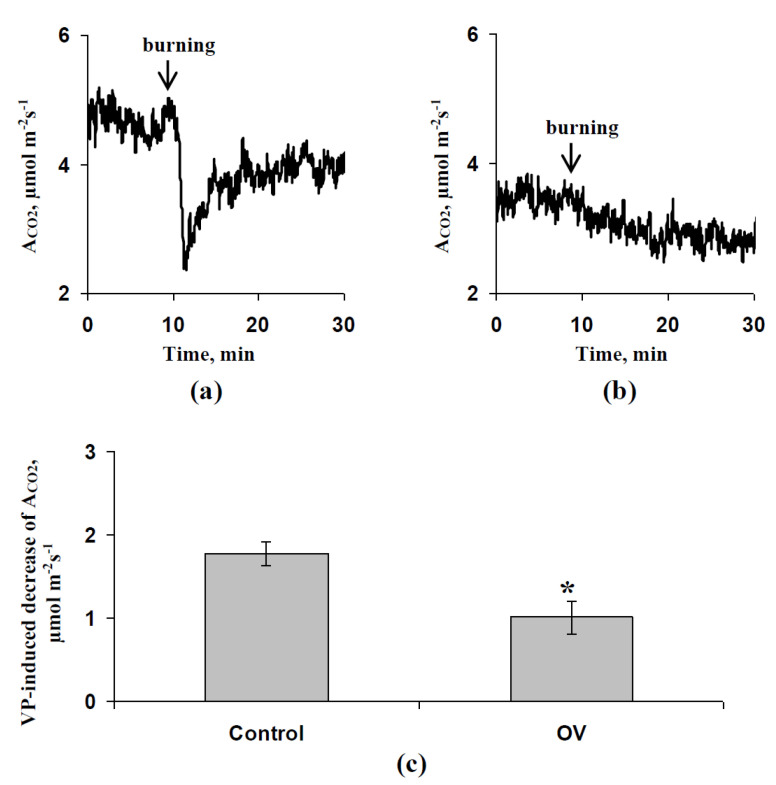
Influence of the modification of H^+^-ATP-ase activity by sodium orthovanadate (OV) on the local burning-induced decreases in CO_2_ assimilation. (**a**) Local burning-induced changes in A_CO2_ in the leaf of a control seedling. (**b**) Local burning-induced changes in A_CO2_ in the leaf of a seedling after vanadate treatment. (**c**) Average magnitudes of local burning-induced A_CO2_ decreases (*n* = 5–6). The preliminary OV treatment of the leaves of seedlings was performed by incubation of the leaf (2 h) in a solution of OV with a moderate concentration (0.5 mM); after that, this leaf was dried by filter paper and photosynthetic measurements were performed. Similar treatment by water was used in the control. Photosynthetic measurements were performed in the second mature leaf; illumination by blue actinic light (239 µmol m^−2^s^−1^) was used. Local burning of the first mature leaf by a flame (3–4 s, about 1 cm^2^) was performed 1.5 h after plant fixation for measurement. *, difference between experiment and control plants is significant (*p* < 0.05).

Figure 10 shows the influence of the decrease in activity of H^+^-ATP-ase in the plasma membrane on the local burning-induced decrease in A_CO2_. It was shown that the preliminary treatment by the moderate OV concentration decreased the magnitude of changes in CO_2_ assimilation, induced by electrical signals; the relative value of this decrease was about 43%. The result supported the hypothesis about the participation of the H^+^-ATP-ase activity decrease in the influence of the exogenous ABA treatment on the regulation of photosynthetic processes by the generation and propagation of electrical signals.

## 4. Discussion

Plant stress hormones, including ABA, play important roles in plant acclimation to the action of adverse factors [1,2]. In particular, it is known that ABA production can be stimulated by drought [3,4], salinization [4,5], nutrient deficiency [6], and different local damages [7,8,9]; the final result of these changes is probably an increase in the plant tolerance to stressors [4,86,87]; in particular, heat tolerance [56,57,58]. From a practical point of view, knowledge of the mechanism of ABA’s influence on physiological processes can be important for creating transgenic plants with an increased stress tolerance or for the development of effective methods for plant treatment by ABA and its analogs under field conditions [14,86,87,88,89].

Photosynthesis is an important target of ABA’s action; in particular, there are numerous works which show the influence of exogenous ABA treatment on photosynthetic processes [28,29,30,31,32,33]. The influence of ABA treatment on photosynthetic processes is considered to be caused by stomata closing [32,34]. However, we previously showed [33] that the spraying of pea and wheat seedlings by water solution with moderate concentrations of exogenous ABA (10^−6^–10^−5^ M) can decrease CO_2_ assimilation, without a significant decrease in stomata conductance; we hypothesized that the effect was caused by a decrease in activity of the H^+^-ATP-ase in the plasma membrane.

The current work highlights some important points.

Treatment (spraying) with a moderate concentration of exogenous ABA (10^−5^ M) decreases the activity of the H^+^-ATP-ase in the plasma membrane, which was estimated on the basis of the metabolic component of the resting potential (Figure 7). This result supports our earlier results and is in good accordance with the work [33].

Treatment (spraying) with a moderate concentration of exogenous ABA decreases the photosynthetic CO_2_ assimilation under light conditions and increases CEF (Figure 2), which supports our earlier results [33]; both processes are linearly correlated. These photosynthetic changes (at least, the A_CO2_ decrease) are probably related to a decrease in the H^+^-ATP-ase activity (Figure 8). This effect is probably not caused by a decrease in the stomata conductance because this conductance is only weakly decreased under treatment of the moderate ABA concentration (10^−6^–10^−5^ M, current work or [33]). This result supports the hypothesis about the participation of the H^+^-ATP-ase activity in the ABA influence on photosynthetic processes, which was proposed in earlier work [33].

Treatment (spraying) with a moderate concentration of the exogenous ABA increases the photosynthetic heat tolerance (the heat tolerance of photosynthetic CO_2_ assimilation) (Figure 3); this effect is probably related to a decrease in the H^+^-ATP-ase activity (Figure 9).

Treatment (spraying) with a moderate concentration of exogenous ABA decreases the amplitude of electrical signals (VP) and magnitude of the A_CO2_ response, induced by these signals (Figure 4); the effect is probably related to a decrease in the H^+^-ATP-ase activity (Figure 10). It is important that a decrease in the photosynthetic CO_2_ assimilation under light conditions is positively related to a decrease in magnitude of the photosynthetic responses (Figure 6).

**Figure 11:**
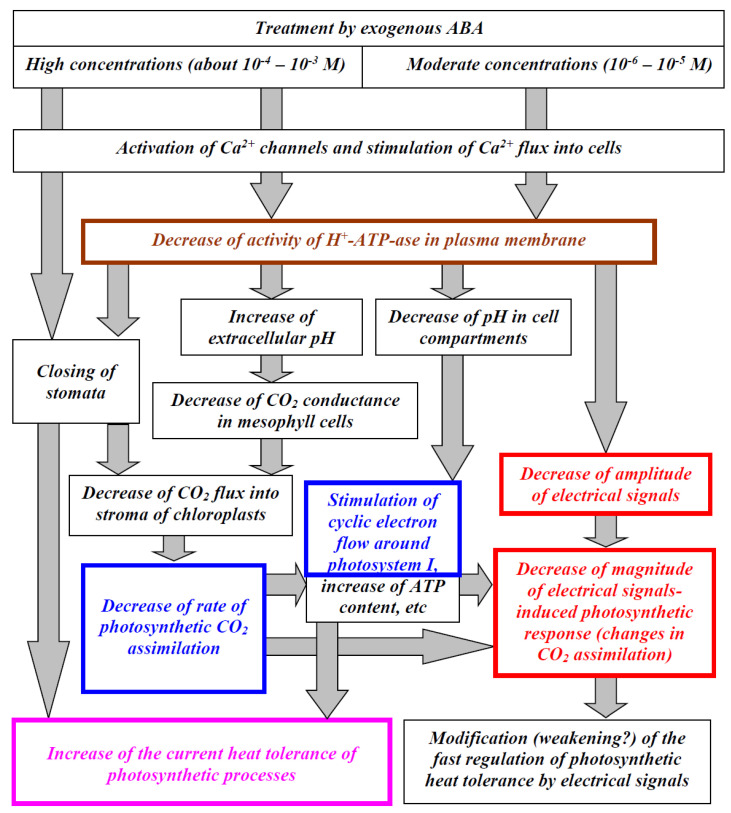
A hypothetical scheme of the potential modes of participation of the H^+^-ATP-ase of the plasma membrane in the influence of the spraying of plants with exogenous ABA on the photosynthetic CO_2_ assimilation, heat tolerance of photosynthetic processes, and their regulation by electrical signals (see text for a detailed description). Colored boxes mark the results which are shown in the current work.

Considering our results and literature data, we proposed a hypothetical scheme of the potential modes of participation of H^+^-ATP-ase of the plasma membrane in the influence of spraying by exogenous ABA on the photosynthetic CO_2_ assimilation, heat tolerance of photosynthetic processes, and their regulation by electrical signals (Figure 11). In accordance with the scheme, spraying with high concentrations (10^−3^–10^−4^ M) of exogenous ABA can induce the closing of stomata and decrease the rate of photosynthetic CO_2_ assimilation [32,86]; this effect is probably caused by the activation of Ca^2+^ channels, which activate S-type anion channels [83]. However, this closing is probably to be weak under moderate concentrations of the exogenous ABA (10^−6^–10^−5^ M, see current work or works [33,90]). Alternatively, exogenous ABA treatment can decrease the activity of the H^+^-ATP-ase in the plasma membrane of leaf cells (see Figure 7 and [12,33,35]); it is probable that this effect is caused by Ca^2+^ influx into cells.

The H^+^-ATP-ase activity decrease can also participate in the stomata closing because its inactivation by treatment of a moderate concentration of sodium orthovanadate decreases the stomata conductance [68]. It is probable that this effect can be related to modification of CO_2_-dependent regulation of stomata [18] because cytokinins (e.g., kinetin), which are antagonists of ABA [15,16], both stimulate stomata opening and produce a positive response to CO_2_ (the stomata opening is greater in ambient than in CO_2_ free air). Moreover, fusicoccin, which is an activator of H^+^-ATP-ase in plasma membrane, induces similar responses: the stomata opening and positive response to CO_2_ [18]. There is a hypothesis [18] that CO_2_ inactivates the plasma membrane H^+^-ATP-ase; i.e., its inactivation by ABA or activation by fusicoccin or kinetin (which can also activate this transporter [91]) should modify CO_2_ influence on stomata and, thereby, change the stomata opening.

It is known that the inactivation of H^+^-ATP-ase can strongly influence the extra- and intracellular pH (e.g., variation potential-induced suppression of its activity induces the alkalization of apoplast and acidification of the cytoplasm, chloroplast stroma, and lumen [38,41,42,43,47,85]); it can be expected that inactivation of the H^+^-ATP-ase induces an increase in the extracellular pH and decrease in the pH in the cell compartments.

An increase in the pH in apoplast decreases the CO_2_ conductance of the plasma membrane [33] and suppresses the photosynthetic CO_2_ assimilation [85,92]. The effect can be caused by pH-dependent changes in the ratio of CO_2_ concentrations to HCO_3_^−^ concentrations in apoplast [44] because the uncharged form can pass through the lipid membranes much easier than the charged one [45,46]. Potentially, other mechanisms may also participate in this suppression of the CO_2_ flux into mesophyll cells (e.g., pH-dependent changes in the activity of aquaporins [41]).

The suppression of photosynthetic CO_2_ assimilation can stimulate a cyclic electron flow around photosystem I (Figure 2d and [33,40]) and increase the ATP content in leaves [93]. As a result, the ABA treatment-induced decrease in CO_2_ assimilation (Figure 2) can stimulate both processes. Moreover, we showed (current work and [33]) that exogenous ABA increased the cyclic electron flow around photosystem I. Activation of the cyclic electron flow [94,95] and an increase in the ATP content [38,54] are known as mechanisms of an increased plant heat tolerance; these mechanisms can be reasons for the ABA treatment-induced increase in the photosynthetic heat tolerance (Figure 3), which is related to the H^+^-ATP-ase activity (Figure 9). It should also be noted that the pH decreases in the cytoplasm, chloroplast stroma, and lumen are known to influence photosynthetic processes [38,42,50,53,85]; it is probable that these decreases can also participate in the induction of photosynthetic changes caused by exogenous ABA treatment.

Additionally, considering the key role of ABA in acclimation to water stress [86,87], it can be expected that the positive influence of the exogenous ABA on the heat tolerance may also be related to stomata closing and a decrease in water loss under heating. In particular, the positive influence of the stomata conductance decrease on the plant heat tolerance was shown in our earlier work [68]; however, this effect can be expected after plant spraying with high concentrations of exogenous ABA.

It is interesting that the changes in photosynthesis, its heat tolerance, and transpiration are similar to responses induced by electrical signals, including a decrease in the CO_2_ assimilation and activation of the cyclic electron flow around photosystem I [42,50,53,59,60,64,65,66,67], increase in the plant heat tolerance [55,68,69,70], and decrease in the stomata conductance [67,68]. This similarity can be explained considering the important role of H^+^-ATP-ase inactivation in the generation of electrical signals (especially, VP [37,40]), in the induction of changes in photosynthesis and its heat tolerance [38], and in the decrease in transpiration [68]. Hypothesizing that the electrical signal-induced photosynthetic response and exogenous ABA treatment-induced one are similar, we should expect a decrease in the photosynthetic response caused by electrical signals after ABA spraying (because the response is already partially formed); the results of the current work support these expectations (the magnitude of the local burning-induced response of A_CO2_ is decreased after the ABA treatment, Figure 5c; this decrease is dependent on the initial rate of photosynthetic CO_2_ assimilation, Figure 6).

However, there is an alternative way of influencing the ABA treatment in the photosynthetic response induced by electrical signals. We showed that the ABA treatment decreases the VP amplitude (Figure 4c and Figure S1c), which is in good accordance with the dependence of VP generation on the H^+^-ATP-ase activity [37]. It is also known that that magnitude of the photosynthetic response is linearly related to the VP amplitude (see works [53,85] and Figure S1d, Supplementary Materials); as a result, the ABA treatment-induced decrease in the VP amplitude can decrease the photosynthetic response.

As a whole, our results show that ABA spraying induces late-onset (1 day after treatment) changes in photosynthetic processes and an increase in the current heat tolerance of photosynthesis; in contrast, the fast photosynthetic regulation by electrical signals (range of minutes) is rather weakened. These findings show that the treatment of plants with exogenous ABA spraying can be potentially used for short-term modification of their tolerance to stressors (e.g., under short-term fluctuation of unstable environmental conditions) and for controlling their electrical activity. Considering the strong relations between plant electrical activity and their physiological processes (including physiological changes induced by the action of environmental factors) [38,40,96,97,98,99,100,101], we suppose that the regulation of electrical activity by ABA treatment can also have potential implications for plant cultivation under increases in short-term environmental changeability.

## 5. Conclusions

The results of our work showed that the treatment of pea seedlings with the spraying of exogenous ABA can influence their photosynthetic parameters (increasing the cyclic electron flow around photosystem I and decreasing the photosynthetic CO_2_ assimilation), increase the photosynthetic heat tolerance, and modify photosynthetic regulation by electrical signals. These effects were related to the activity of the H^+^-ATP-ase in the plasma membrane; moreover, the exogenous ABA treatment decreased this activity. It can be supposed that the ABA treatment-induced decrease in the H^+^-ATP-ase activity is a potential way of influencing of the exogenous ABA spraying on photosynthetic processes, their heat tolerance, and regulation by electrical signals. As a whole, our results can be potentially useful for development of new methods of management of plant tolerance in agriculture (on the basis of the exogenous ABA treatment or by using other methods for modification of H^+^-ATP-ase activity in the plasma membrane).

## Supplementary Materials

The following are available online at https://www.mdpi.com/2079-7737/9/10/324/s1, Figure S1: (a) Extracellular record of burning-induced variation potential (VP) in leaf of the control pea seedling. (b) Extracellular record of burning-induced variation potential (VP) in leaf of the ABA-treated seedling. (c) Average amplitudes of VP (AVP) in control seedlings and seedlings in 1 day after the ABA treatment at extracellular measurements (*n* = 5–7). (d) The scatter plot between amplitudes of VP at extracellular measurements and magnitudes of VP induced decreases in A_CO2_ in pea seedlings (ΔA_CO2_) (*n* = 12). Figure S2: Examples of measurements of the metabolic component of the resting potential in control (a), ABA-treated (b) and preliminarily OV-treated (c) pea seedlings.
