# Supplementary material for: Exogenous Abscisic Acid Can Influence Photosynthetic Processes in Peas through a Decrease in Activity of H+-ATP-ase in the Plasma Membrane
Source: Biology (Basel). 2020 Oct 4;9(10):324. doi: 10.3390/biology9100324 (PMC7650568; doi:10.3390/biology9100324)
Supplement: Supplementary file 1 [file biology-09-00324-s001.zip › Fig.S1.pdf]

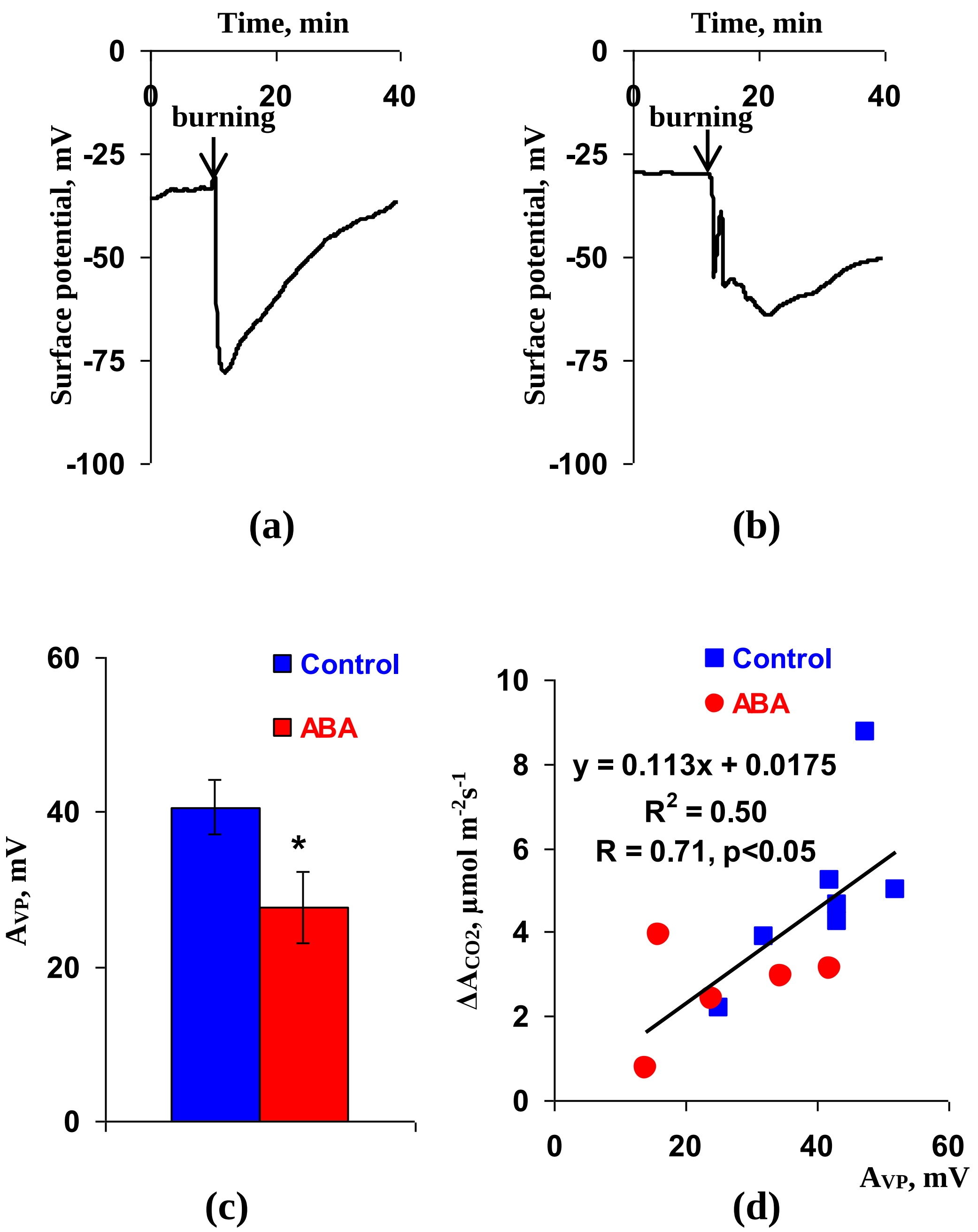

Figure S1. (**a**) Extracellular record of burning-induced variation potential (VP) in leaf of the control pea seedling. (**b**) Extracellular record of burning-induced variation potential (VP) in leaf of the ABA-treated seedling. (**c**) Average amplitudes of VP (A_{VP}) in control seedlings and seedlings in 1 day after the ABA treatment at extracellular measurements (*n*=5-7). (**d**) The scatter plot between amplitudes of VP at extracellular measurements and magnitudes of VP-induced decreases in A_{CO2} in pea seedlings (ΔA_{CO2}) (*n*=12). The ABA treatment of seedlings was performed by spraying with its aqueous solutions (10^{-5} M) in 1 day before electrical measurements; control plants were treated with equal volumes of water. Variation potentials were induced (burning of the first mature leaf by flame, 3-4 s, about 1 cm^2) after 1.5 h of adaptation. Electrical measurements were performed in the second mature leaf. A_{VP} was calculated as difference between initial and minimal values of the surface potential. Negative direction of changes in surface potential at extracellular measurements was in accordance with positive direction of changes in the membrane potential at microelectrode measurements. *, difference between experiment and control plants is significant ($p<0.05$). R^2 and R are determination and correlation coefficients, respectively.
