# Supplementary material for: Exogenous Abscisic Acid Can Influence Photosynthetic Processes in Peas through a Decrease in Activity of H+-ATP-ase in the Plasma Membrane
Source: Biology (Basel). 2020 Oct 4;9(10):324. doi: 10.3390/biology9100324 (PMC7650568; doi:10.3390/biology9100324)
Supplement: Supplementary file 1 [file biology-09-00324-s001.zip › Fig.S2.pdf]

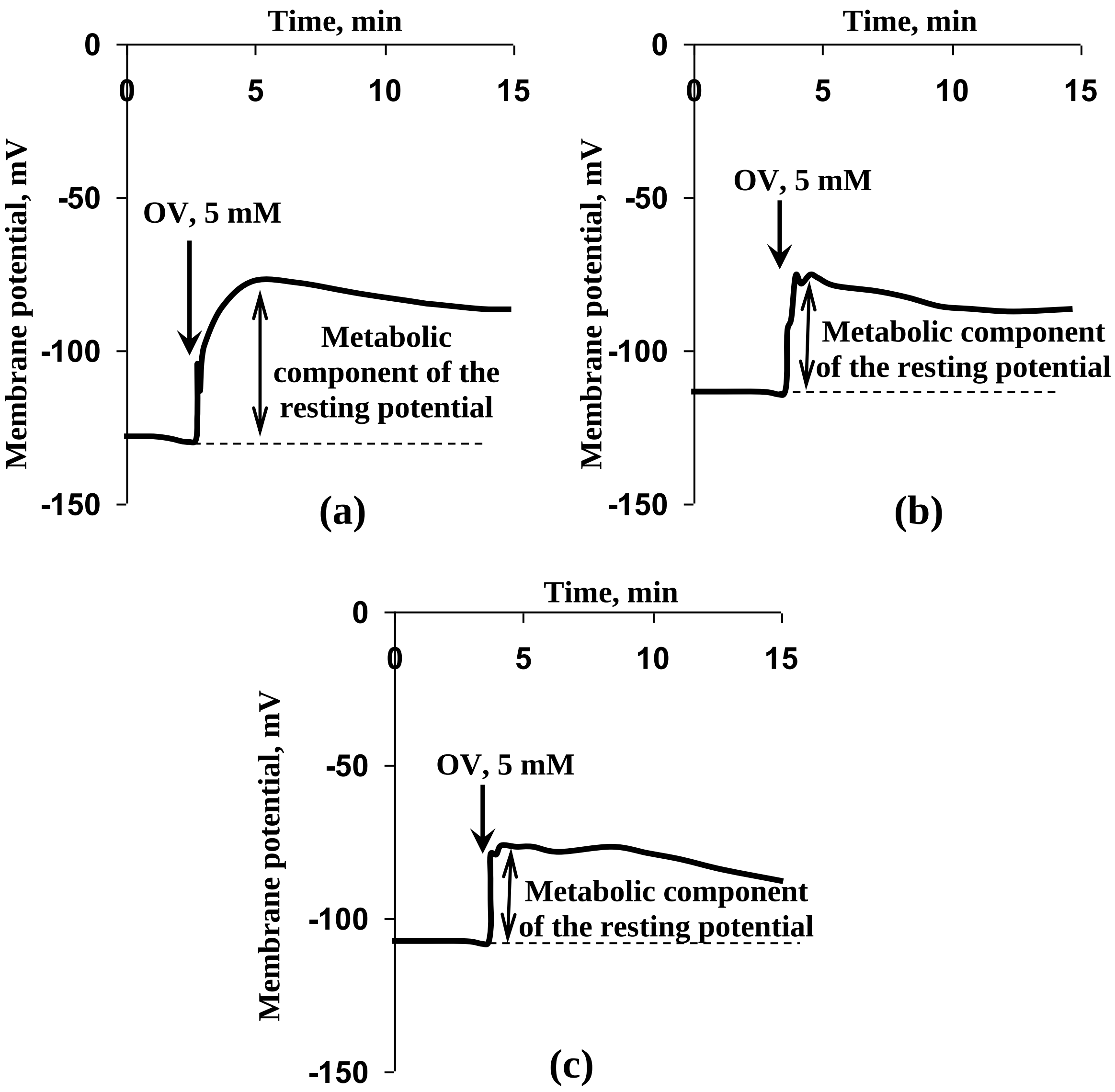

Figure S2. Examples of measurements of the metabolic component of the resting potential in control (a), ABA-treated (b) and preliminarily OV-treated (c) pea seedlings. The ABA treatment of seedlings was performed by spraying with its aqueous solutions (10^{-5} M) in 1 day before electrical measurements; control plants were treated with equal volumes of water. The preliminary treatment by the moderate OV concentration in the second mature leaf in seedlings was performed by incubation of the leaf (2 h) in solution of OV (0.5 mM); after that this leaf was dried by filter paper and was used for measurement of electrical activity. Similar treatment by water was used in control. Measurements of the metabolic component of the resting potential were performed with addition of high concentration of OV (5 mM) during the electrical record; only, short-term changes in membrane potential were analyzed.
